# A comparison of surgical approaches in the treatment of grade C postoperative pancreatic fistula: A retrospective study

**DOI:** 10.3389/fsurg.2022.927737

**Published:** 2022-08-09

**Authors:** Pavel Záruba, Michael Rousek, Tereza Kočišová, Karolína Havlová, Miroslav Ryska, Radek Pohnán

**Affiliations:** Department of Surgery, 2nd Faculty of Medicine of Charles University and Military University Hospital Prague, Prague, Czechia

**Keywords:** pancreatic fistula, POPF, pancreas, pancreaticoduodenectomy, pancreatic resection

## Abstract

**Background:**

Postoperative pancreatic fistula is one of the most dreaded complications following pancreatic resections with Grade C the most severe. Several possible types of surgical intervention are available but to date, none of them have clearly shown superiority. This study aims to compare different surgical approaches.

**Methods:**

A retrospective analysis of patients who underwent revision surgery for postoperative pancreatic fistula between 2008 and 2020 was performed. Three surgical approaches were compared: open drainage; a disconnection of the pancreaticojejunostomy; and salvage total pancreatectomy. The data of nine monitored parameters were collected. Selected parameters were statistically analyzed and compared.

**Results:**

A total of 54 patients were included. Eighteen patients underwent open drainage, 28 had disconnections of the pancreaticojejunostomy and eight had salvage total pancreatectomy. Statistically significant differences were observed in the time of Intensive Care Unit stay, the number of surgical interventions, 90-day mortality, the number of administered blood transfers and treatment costs. Open drainage showed to be superior in each category. The difference in long-term survival also slightly favored simple drainage.

**Conclusion:**

Open drainage procedure showed to be superior to other types of interventions in most of the monitored parameters. Disconnection of the pancreaticojejunostomy and a salvage total pancreatectomy had similar results, which correlated with the surgical burden of these interventions.

## Introduction

A postoperative pancreatic fistula (POPF) is one of the most severe complications after pancreaticoduodenectomy (PDE). It is defined as the abnormal communication between the pancreatic ductal epithelium and another epithelial surface containing pancreas-derived, enzyme-rich fluid. The definition and classification of POPF were established by the International Study Group of Pancreatic Fistula (ISGPS) (1). In the last revision from 2016, the perception of the least severe type, previously POPF A, was revised. The definition of POPF grades B and C were modified (2). Grade C is the most severe type of POPF. It is characterized by either multiorgan failure, the need for revision surgery or death directly associated with the presence of the fistula. It remains a serious clinical problem, mainly due to poor treatment results and high mortality (3, 4). Prolonged intensive care unit stays, repeated surgical interventions, and increased economic burden are all directly associated with grade C POPF (5). The subsequent decline in overall patient performance status, poor quality of life and prolonged recovery often lead to withdrawal of potential further oncological treatment (6, 7). Grade C POPF almost always requires re-laparotomy with the intention of deriving leaking pancreatic juice from insufficient pancreaticojejunal anastomosis (PJA). Several possible surgical approaches exist to date. However, none have proven clear superiority (8–10). The strategy of treatment is commonly chosen individually, depending on radiological or perioperative findings, a patient's condition and personal experience. Open simple drainage of the insufficient anastomosis is technically less demanding and less invasive for the patient (11). Salvage total pancreatectomy, regarded as a burdensome intervention with high perioperative mortality is an alternative (12). Various studies addressed this problem leading to different treatment preferences. Pancreas preserving strategies are commonly favored (13–15). Most studies focusing on surgical treatment of Grade C postoperative pancreatic fistula are of small groups of highly selected patients. The results show a wide variation of operative outcomes across studies. A comparative analysis between surgical treatment approaches across the studies is methodologically not always feasible (16). This work compares the results of different surgical strategies for treating grade C pancreatic fistula in a single-center retrospective analysis.

## Material and methods

The prospectively collected data from our electronic database were retrospectively evaluated. The data of 682 patients who underwent PDE in the 2008–2020 period for benign or malignant affection of the head of the pancreas, duodenum, or terminal portion of the common bile duct were analyzed. Patients with revision surgery for POPF C were included in this study. The baseline characteristics of all patients who underwent the PDE during the 2008–2020 period are shown in Table 1.

**Table 1:** Baseline characteristics of the patients who underwent the PDE in the 2008–2020 period.

PDE		PC	CPL	CBD	DAT	NET	CHP	others	All
	N	307	87	42	74	34	117	21	682
% of performed PDE	%	45	12.8	6.1	10.8	4.9	17.2	3.2	
Gender	M/F	0.5/0.5	0.4/0.6	0.8/0.2	0.7/0.3	0.3/0.7	0.8/0.2	0.5/0.5	0.6/0.4
Average age		65	63	64	62	55	54	63	62
Whipple	N	76	29	8	28	15	35	10	201
PPPDE	N	179	55	30	46	17	78	7	412
Venous resection	N	52	3	4	0	2	4	4	69
CD III + IV	N	92	34	19	29	14	25	12	225
	%	30.1	39.5	45.2	38.7	41.3	21.4	53.5	33
POPF B	N	17	8	7	7	5	7	2	52
	%	5.5	9.6	16	9.1	15	5.7	7.7	7.6
POPF C	N	14	10	7	10	4	5	4	54
	%	4.6	11.5	16.7	13.6	11.9	4.3	18.4	7.9
30-day mortality	N	15	3	3	8	1	3	3	37
	%	4.9	3.8	8	11	3	2.8	15	5.5
90-day mortality	N	27	12	5	11	1	3	5	64
	%	8.7	13.4	12	15	3	2.8	23	9.3

PC, pancreatic cancer; PCL, cystic pancreatic lesion; CBD, common bile duct carcinoma; DAT, duodenal and ampullary tumor; NET, neuroendocrine tumor; CHP, chronic pancreatitis. Others stay for rare indications for PDE as metastases to the pancreas, multiorgan resections, GIST etc. CD III + IV: postoperative complication Clavien-Dindo classification grade III + IV, PPPDE: pylorus preserving pancreatoduodenectomy.

### Surgical method

Both pylorus preserving pancreaticoduodenectomy (Traverso-Longmire) and Whipple procedures were performed. A total of four surgeons from one pancreatic team performed all pancreatic resections. Standard antibiotic prophylaxis was used. The reconstruction of the supramesocolic area was usually performed simultaneously on one loop of the oral jejunum in a specific order: 1. pancreaticojejunal anastomosis (PJA), 2. hepaticojejunal anastomosis, 3. gastrointestinal anastomosis. The PJA was constructed as one-layer end to side anastomosis with single monofilament absorbable 3/0 or 4/0 stitches. The fiber diameter was chosen based on the texture of the pancreatic tissue. A nasojejunal tube was routinely placed along with a nasogastric tube. Finally, each patient had abdominal drainage placed nearby the PJA. In addition to standard laboratory monitoring, including serum amylase activity, we routinely measured drain amylases on the first, third and fifth postoperative days. The ISGPF's criteria were used in the diagnostics of POPF (2). In case of clinical deterioration of patients with POPF, computed tomography (CT) scan was performed. Septic shock, the development of organ failure, or a collection unsuitable for CT guided drainage were indications for re-laparotomy. The local findings, the extent of PJA dehiscence and the severity of organ dysfunction with overall patient status were all considered when choosing a surgical strategy. One of three types of intervention were used:

A re-laparotomy with extension drainage of the insufficient pancreaticojejunal anastomosis (PJA) was used if only a minor portion was dehiscent. (*Simple drainage group*).

Disconnection of PJA along with drainage of the pancreatic stump was performed when most of the PJA failed (*PJA disconnection group*). The pancreatic juice leakage led to retention of fluid around PJA causing local peritonitis. In this technique the pancreaticojejunostomy was taken down. The blind jejunal end was resected with a stapler or hand-sewn closure was performed. Additional drainage was placed near the pancreatic resection line. A small caliber catheter can be inserted into the main pancreatic duct as an external wirsungostomy. A minority of patients may eventually develop a pancreatic pseudocyst in the near future. In these cases, an endoscopic pancreaticopseudocystostomy is usually feasible. However, according to our experience, in most cases no further intervention is needed.

A salvage total pancreatectomy with splenectomy was chosen if the PJA leakage was associated with the necrosis of the pancreatic remnant (*Total pancreatectomy group*).

### Data collection

Depending on the chosen surgical strategy type, patients were divided into three subgroups. There were collected data on the total length of the hospital stay, the length of the Intensive Care Unit-stay, the total number of surgical interventions, the duration of antibiotic therapy, the count of administered blood transfers, direct financial costs and the length of use of the negative pressure wound therapy (NPWT). The 30-day and 90-day mortality were monitored. The data on long-term survival were collected. Financial costs data were obtained from the electronic database. The information about a patient's condition, provided therapy and specific procedures were collected. Each procedure has its own specific code which serves for the payment and communication with the insurance companies in Czechia. All insurance companies in Czechia cover the provided health care in the same way.

### Statistical analysis

Statistical analysis was performed using the Program STATISTICA 13.2 (Tibco software). Kruskal-Wallis ANOVA was used to compare three groups. Mann-Whitney tests were used to compare differences between 2 groups. Correlations were evaluated using Spearman's ordinal coefficient. Kaplan-Meier analysis with log-rank tests was used to compare survival between groups. The p-value of 0.05 was considered statistically significant.

## Results

A total of 54 patients out of 682 pancreaticoduodenectomies developed a grade C postoperative pancreatic fistula (7.9%). The overall incidence of a grade B and grade C fistula was 15.5%. The mean age was 63 years (25–83). All patients underwent a re-laparotomy. The monitored parameters of the three groups are shown in Table 2. Disconnection of the pancreaticojejunostomy with a blind closure of the jejunal loop and pancreatic stump drainage was performed in 28 cases (52%). Simple drainage of insufficient PJA was performed in 18 patients (33%). Eight patients (15%) underwent a total salvage pancreatectomy. Seven patients underwent a CT-guided drainage, endovascular intervention or endoscopic intervention before the re-laparotomy. In 8 patients, a stepwise approach was applied. In seven out of eight patients, a disconnection of PJA after open drainage failure was performed. In one patient, a completion of a total pancreatectomy because of PJA disconnection failure was performed. The mortality in this subgroup was 63% (5 out of 8 patients). The average time from PDE to the first re-laparotomy was 5.4 days (2–11) in the Disconnection group, 12.2 days (1–40) in the Open drainage group, and 4.9 days (1–12) in the Total pancreatectomy group.

**Table 2:** Monitored data of the patients with Grade C postoperative pancreatic fistula indicated for re-laparotomy.

	Disconnection of PJA	Open drainage	Total pancreatectomy	*p*-value
Number of cases	28 (52%)	18 (33%)	8 (15%)	* *
Time to re-laparotomy (days)	5.4 (2–11)	12.2 (1–40)	4.9 (1–12)	
Hospital stay (days)	41.6 (14–82)	39.4 (19–107)	33.1 (13–77)	*p* = 0.225
ICU stay (days)	22.4 (2–57)	10.3 (0–45)	23.6 (5–74)	*p* = 0.043
Number of re-laparotomies (*n*)	6.7 (1–17)	2.9 (1–10)	5.6 (1–12)	*p* = 0.002
Length of antibiotic treatment (days)	28.1 (6–50)	21.4 (0–40)	21.8 (1–43)	*p* = 0.337
Administered blood transfusions (units)	12.2 (1–58)	3 (0–10)	9.8 (2–15)	*p* = 0.018
Total costs per patient (thousand of Euros)	37.3 (13.3–80.3)	18.8 (6.1–32.2)	30.2 (13.3–65.7)	*p* = 0.004
Duration of NPWT treatment (days)	11.1 (0–40)	3.9 (0–19)	8.9 (0–24)	*p* = 0.058
30-day mortality	18% (*n* = 5)	6% (*n* = 1)	25% (*n* = 2)	*p* = 0.352
90-day mortality	46% (*n* = 13)	6% (*n* = 1)	38% (*n* = 3)	*p* = 0.013

**Figure 1:**
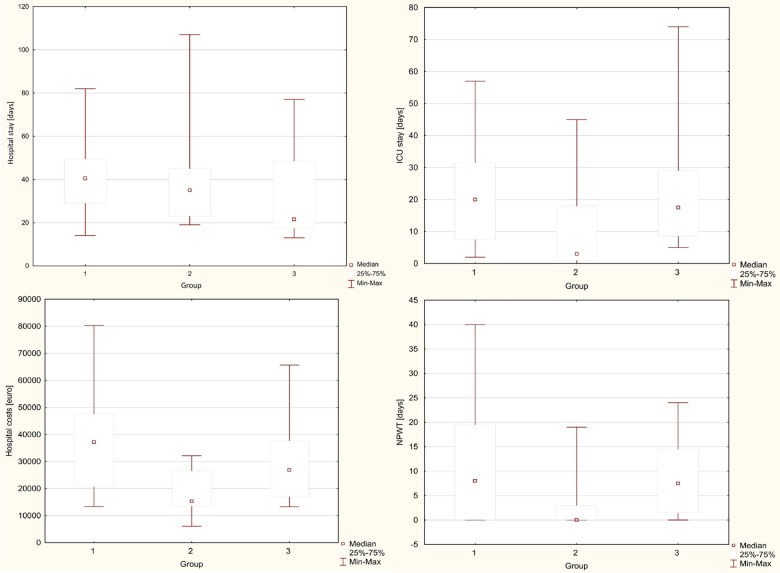
Boxplots comparing the individual monitored parameters between the groups. Group 1: PJA disconnection group; Group 2: Simple drainage group; Group 3: Total pancreatectomy group.

**Figure 2:**
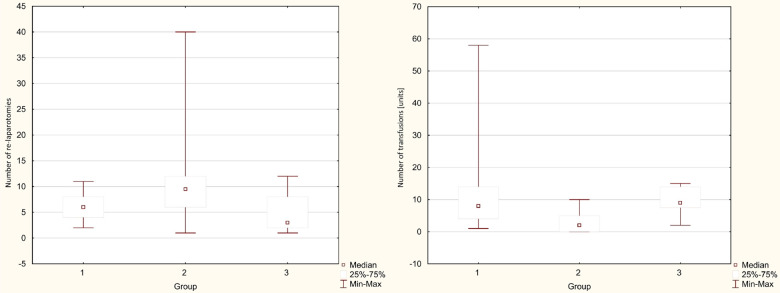
Boxplots comparing the individual monitored parameters between the groups. Group 1: PJA disconnection group; Group 2: Simple drainage group; Group 3: Total pancreatectomy group.

The average length of hospital stay in individual groups was 41.6 vs. 39.4 vs. 33.1 days (13–107), with no statistical difference between the groups (*p* = 0.225). The average Intensive Care Unit stay length was 22.4 vs. 10.3 vs. 23.6 days (0–74), with a significantly shorter time in the Simple drainage group (*p* = 0.043). The average number of the subsequent surgical interventions was 6.7 vs. 2.9 vs. 5.6 (1–17), with a statistically significant difference between the groups (*p* = 0.002) in favor of the Simple drainage group. A statistically significant difference favoring the Simple drainage group was also found in the need for blood transfers (*p* = 0.018). The average number of given blood units was 12.2 vs. three vs. 9.8. The treatment costs per patient were 37.3 vs. 18.8 vs. 30.2 thousand Euros per patient, and there was also a significant difference favoring the Simple drainage group. (*p* = 0.008). The average duration of NWPT was 11.1 vs. 3.9 vs. 8.9 days. The difference in the time of the NPWT use was marginally statistically significant (*p* = 0.058). It strongly correlated with the number of surgical revisions. The individual monitored parameters between the groups are shown in boxplots in Figures 1, 2.

The duration of the antibiotic therapy was comparable between the groups (*p* = 0.337). The average length of antibiotic treatment was 28.1 vs. 21.4 vs. 21.8 days. The 30 and 90-day mortality of patients with POPF C was 15% and 31%, respectively. The 30-day mortality was 18% vs. 6% vs. 25% in individual groups, and there was no statistical difference (*p* = 0.352). In contrast, the 90-day mortality was significantly lower in the Simple drainage group. The 90-day mortality was 46% vs. 6% vs. 38% in the PJA disconnection group, the Simple drainage group and the Total pancreatectomy group, respectively (*p* = 0.013).

**Figure 3:**
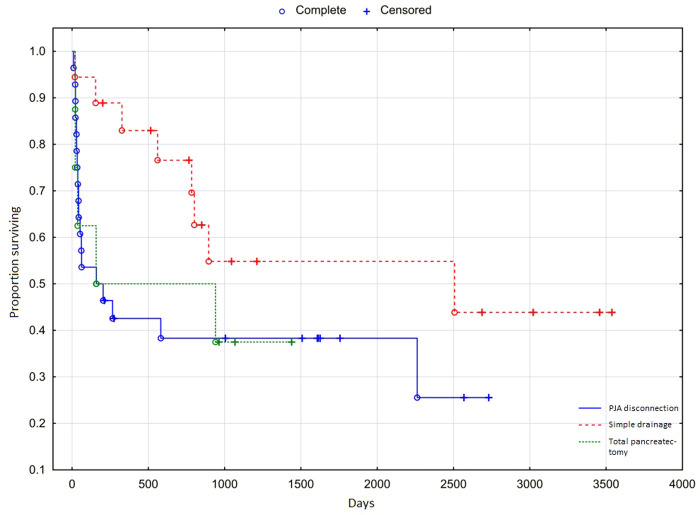
Overall survival of patients with Grade C postoperative pancreatic fistula.

The median survival of patients with POPF C was 789 days (26 months). Figure 3 shows the overall survival curves for individual subgroups. The PJA disconnection group and the Total pancreatectomy group have almost identical courses, with a slightly different curve course representing the Simple drainage group. However, the difference was below the limit of statistical significance (*p* = 0.068).

## Discussion

A recent exhaustive review of more than 60,000 patients showed the incidence of postoperative pancreatic fistula in 21.3% of the patients after pancreaticoduodenectomy (17). The most severe type, grade C, is fortunately infrequent. The incidence of Grade C POPF in the meta-analysis was 3.5%. It varied from less than 1% to more than 9% (17). The mortality of POPF C is high, with the described incidence rate of 25%–35% (17, 18). Also, in our cohort of 54 patients with the Grade C postoperative pancreatic fistula, the mortality rate was 31%. Most patients with POPF C need at least one surgical re-intervention (19). According to the published literature, the surgical treatment strategies are considerably varied, and no surgical strategy showed clear superiority. In the analysis of 24 studies containing 370 patients, the frequency of individual approaches was calculated. Total pancreatectomy was performed in 200 cases (54.1%), PJA disconnection with preservation of the pancreas in 47 (12.7%), internal or external wirsungostomy in 67 (18.1%), pancreaticogastrostomy in 8 (2.2%) and simple PJA drainage in 48 (12.9%). The overall mortality rate stated in this paper was 34.3%. After completing a total pancreatectomy, the mortality rate was 42%, and the endocrine pancreatic insufficiency was naturally 100%. A completion of a total pancreatectomy was considered a burdensome event with a high mortality (18).

The most common indications for completion of pancreatectomy are peritonitis and sepsis, followed by bleeding and pancreatitis of the pancreatic remnant (16, 20). In our study a salvage total pancreatectomy was performed in 8 (15%) patients. Pancreas-preserving interventions were performed in 85% of cases. The PJA disconnection with jejunal closure and pancreatic stump drainage was clearly favored. It was performed in 52% of the cases. In the recently published single-center retrospective study, no significant difference in mortality was found in comparing simple drainage, PJA disconnection and total pancreatectomy (21). Also, no significant difference in mortality was found in the study comparing resuture of the PJA, renewal of the anastomosis, and completion of pancreatectomy (22). Similarly, the 30-day mortality rate showed no statistical significance between the groups in our study. However, the 90-day mortality showed the Simple drainage group superiority. Moreover, the Simple drainage was significantly superior in most evaluated parameters. The statistically significant differences in favor of the Simple drainage group were observed in the time of Intensive Care Unit stay, the need for administered blood transfers and treatment costs. Contrary to our expectations, no significant difference in the length of hospital stay was found. The total number of the subsequent surgical interventions also favored a Simple drainage group. However, the results may be distorted by the indications for the subsequent re-intervention. In the Total pancreatectomy group, the indication for re-intervention was a management of the NPWT in 6 of 7 patients. Also, at least one re-laparotomy was necessary for all patients within the PJA disconnection group due to NPWT use. This finding supports the well-recognized correlation between NPWT length of use and the number of subsequent laparotomies.

The presented data showed that simple drainage might lower the surgical burden and bring subsequent better results in patients requiring re-laparotomy for postoperative pancreatic fistula.A stepwise approach, i.e. PJA disconnection or completion of pancreatectomy after open drainage failure, or salvage total pancreatectomy after a PJA disconnection failure is a treatment option for these patients. However, in our experience, the results are not encouraging. Patients are usually in a prolonged critical condition, and the procedure could be technically exceptionally challenging. The mortality was 63% (5 out of 8 patients) in our study. Extremely poor results and high mortality in patients after the completion of pancreatectomy because of an open drainage failure are described by other authors as well (21). Our study also confirmed that the total pancreatectomy is associated with known high morbidity and mortality. Surprisingly, our analysis showed similarly unfavourable results in the PJA disconnection group. The results of monitored parameters of the PJA disconnection group are comparable to the Total pancreatectomy group. However, during the long-term follow-up, the patients after PJA disconnection had a distinctly lower incidence of pancreatic endocrine insufficiency compared to patients with total pancreatectomy (18).

It should be noted that our study has some limitations. It is a monocentric, retrospective study. The group of included patients is heterogeneous, and multivariate analysis was not feasible. The indications for the surgical approaches were made individually, according to the actual status of the patient and local findings. Due to the relative rarity of grade C POPF, a long time interval of data collection has to be chosen. This may imply a non-negligible risk of bias. However, a pancreaticoduodenectomy and specific treatment approach to a postoperative complication were always performed with the same pancreatic team.

Our conclusions are consistent with a recently published single-center study, suggesting simple drainage as the most suitable method for severe postoperative pancreatic fistula treatment (23).

## Conclusion

Grade C POPF represents the most severe form of the postoperative pancreatic fistula. The treatment strategy has not been unified, and no method has proven to be clearly superior. The results of our study support the simple drainage of the leaking PJA as a method of choice. This strategy is the least invasive and the least demanding intervention. However, it is not generally applicable. The PJA disconnection and the total salvage pancreatectomy may be reserved for specific situations considering a significant burden of these interventions.

## Data Availability

The raw data supporting the conclusions of this article will be made available by the authors, without undue reservation.
